# Functional outcomes after transoral CO_2_ laser treatment for posterior glottic stenosis: a bicentric case series

**DOI:** 10.1007/s00405-022-07516-2

**Published:** 2022-07-10

**Authors:** Marta Filauro, Francesco Missale, Alberto Vallin, Francesco Mora, Valeria Marrosu, Filippo Carta, Roberto Puxeddu, Giorgio Peretti

**Affiliations:** 1 IRCCS Ospedale Policlinico San Martino, 16132 Genoa, Italy; 2 Unit of Otorhinolaryngology, Head and Neck Surgery, Department of Surgical Sciences and Integrated Diagnostics (DISC), University of Genoa, Largo Rosanna Benzi, 16132 Genoa, Italy; 3 Department of Experimental Medicine (DIMES), University of Genoa, Genoa, Italy; 4 Department of Molecular and Translational Medicine, University of Brescia, 25121 Brescia, Italy; 5 Department of Head & Neck Oncology & Surgery Otorhinolaryngology, Antoni Van Leeuwenhoek, Nederlands Kanker Instituut, 1066 Amsterdam, The Netherlands; 6 Department of Otorhinolaryngology-Head and Neck Surgery, University of Cagliari, 09124 Cagliari, Italy

**Keywords:** Posterior glottic stenosis, Transoral CO_2_ laser treatment, Bogdasarian, TOLMS, Posterior cordotomy, Mucosal microflap, Functional outcomes, Interarytenoid, Synechia, Cricoarytenoid joint, Endoscopic lysis

## Abstract

**Purpose:**

The aim of this study is to evaluate functional outcomes in terms of decannulation rate and quality of life of patients affected by PGS (Grades I–IV) treated only by transoral CO_2_ laser microsurgery (TOLMS) in two tertiary centers.

**Methods:**

An observational retrospective study was carried out, enrolling 22 patients affected by PGS who were treated by a transoral approach at two tertiary referral centers. Surgical treatment included TOLMS with tailored laser resection of the scar tissue combined with posterior cordotomy, resurfacing of the raw area with mucosal microflap, or placement of a Montgomery T-tube or Keel stent. All patients were evaluated and staged preoperatively and postoperatively, at least 6 months after the surgery. Functional outcomes were objectively evaluated by the Airway-Dysphonia-Voice-Swallowing (ADVS) staging system, Voice Handicap Index-30 (VHI-30), and Eating Assessment Tool-10 (EAT-10) questionnaires.

**Results:**

Quality of life significantly improved as measured by the VHI-30 questionnaire with a median variation of − 31.0 (*p* = 0.003), the EAT-10 with a median variation of − 4.0 (*p* = 0.042), and the ADVS with a median variation of − 3.5 (*p* < 0.001). No significant changes were observed in swallowing scores. We were able to decannulate 7 of 9 patients (almost 80%) with previous tracheotomy.

**Conclusion:**

In conclusion, even if there is still no general agreement on an exact therapeutic algorithm to treat PGS, our results confirm that transoral surgery, in terms of scar tissue removal, combined in selected patients with posterior cordotomy and pedicled local flaps and/or placement of stents, represents a safe and effective surgical approach even for more severe PGS.

## Introduction

Posterior glottic stenosis (PGS) is a critical pathological condition in which the vocal folds are fixed in a phonation position. This results in a severe reduction of airway patency at the posterior glottic area, which is of paramount importance during respiration accounting for 50–60% of the entire airway glottic lumen [1, 2]. Scar tissue formation is responsible for PGS, developing in the interarytenoid area and potentially going as far as the arytenoids and cricoarytenoid joints. This may cause uni- or bilateral impairment and/or fixation of the arytenoid. The most common etiology of PGS is prolonged and/or traumatic intubation. Indeed, a direct correlation between duration of orotracheal intubation and the incidence of PGS has been reported, occurring in 5% of patients intubated for 5–10 days and in 12% of patients intubated for 11–24 days [3]. The traumatic pressure of the endotracheal tube on the posterior commissure is the most common cause of mucosal ulceration, chondritis, prolonged inflammation with exuberant granulation tissue formation, and consequential fibrosis that extends to the arytenoid and, finally, to the cricoarytenoid joint [2]. More rarely, PGS can be caused by other factors, such as infection, external trauma, laryngopharyngeal reflux, inhalation, caustic ingestion, and previous surgery or radiotherapy [4].

The clinical presentation of PGS is similar to bilateral vocal fold paralysis (BVFP): patients usually complain of dyspnea and stridor without a significant dysphonia since the vocal folds in a paramedian position in both conditions. Since the etiology, chances of spontaneous recovery, and treatment of PGS and BVFP are different, accurate differential diagnosis is mandatory. Awake flexible endoscopy evaluation and palpation of cricoarytenoid joints in microlaryngoscopy under general anesthesia may be essential for differential diagnosis. In the last decades, several authors [5, 6] have suggested that laryngeal electromyography (LEMG) should also be used for to fine-tune differential diagnosis of laryngeal neurologic disorders, PGS, and cricoarytenoid joints disorders.

The choice of surgical treatment is strictly associated with local variables, such as the type, degree, and vertical extension of the stenosis. Moreover, comorbidities, patient age, etiology of stenosis, and the experience of the airway team all play key roles in the decision-making process.

Bogdasarian et al.[7] categorized PGS into four grades based on the progressively greater involvement of the posterior commissure and cricoarytenoid joints: grade I interarytenoid synechia with a sinus tract posteriorly, grade II interarytenoid and posterior commissure scarring, and Grade III involves a posterior commissure scar or web with fixation of one cricoarytenoid joint. Grade IV, the most challenging to treat, is characterized by the fixation of both cricoarytenoid joints.

Careful examination of the remainder of the airway is necessary to detect other concomitant airway abnormalities. The extension to the subglottic and tracheobronchomalacia is frequently associated with PGS and has a strong impact on its management [8]. Younger patients have a greater risk of multilevel airway stenosis involving both the subglottis and trachea, which makes reconstruction more demanding and leads to poorer outcomes compared to those with PGS localized at the glottic level [1].

It is widely recognized that the first choice of treatment for mild PGS (Grades I–II) is endoscopic lysis, since healthy tissue, spared between the row areas due to the resection of the fibrotic bridge, avoids web reformation in the interarytenoid region. For patients affect by more severe PGS, mere lysis of the synechia typically leads to scar recreation and recurrence. For this reason, in patients affected by more severe PGS (Grades III–IV), elaborate laryngoplasty procedures are needed [9–11]. Actually, several surgical techniques have been shown to avoid PGS recurrence, such as the postcricoid mucosal advancement flap (PMAF), placement of a keel or a stent, and the interposition of a cartilaginous graft between the split cricoarytenoid units [4, 12–14]. The vast majority of these surgical procedures can be performed transorally, while an open-neck approach is reserved for more complex cases. Transoral CO_2_ laser microsurgery (TOLMS) is strongly recommended for PGS Grades I–II [3, 15]. Especially for PGS grade II, TOLMS is a valuable option in combination with PMAF [1, 4]. On the other hand, for more severe PGS (Grades III and IV) a number of authors strongly recommend airway expansion, especially if associated with subglottic stenosis [16–18].

The aim of the present study is to evaluate functional outcomes in terms of decannulation rate and quality of life of patients affected by PGS (Grades I–IV) treated only by TOLMS in two tertiary referral centers.

## Materials and methods

An observational retrospective study was carried out, enrolling 22 patients affected by PGS who were treated with a transoral approach at the Otolaryngology—Head and Neck Surgery Unit of the IRCCS Ospedale Policlinico San Martino of Genoa, Italy, and the Otolaryngology—Head and Neck Surgery Unit of the University Hospital of Cagliari, Italy between January 2013 and December 2020 (CER Liguria: 63/2021—DB id 11,240). Thirteen (59%) patients were males and 9 (41%) were females (male to female ratio, 1.44 to 1), with a mean age of 55 years (range 11–85). Twelve (55.5%) were already tracheostomized at the first consultation. The etiology of the stenosis was iatrogenic in 18 (82%): post-actinic in 9 (41%), post-intubation in 5 (23%), and caused by previous surgery in 4 (18%). In the remaining 4 (18%) cases, the PGS was correlated with trauma in 1 (4.5%), autoimmune diseases in 1 (4.5%), and idiopathic in 2 patients (9%) (Table 1). All patients underwent routine diagnostic work-up, including awake flexible videolaryngostroboscopy (Kay Pentax Laryngeal Strobe 9400; Pentax Medical, Montvale, NJ, USA) combined with panendoscopy under general anesthesia with 0° and 70° rigid telescopes using a HDTV system (Olympus Medical System Corporation, Tokyo, Japan). The grade of stenosis was assessed according to the Bogdasarian classification [7]. According to this classification, 2 (9%) patients were affected by grade I, 3 (14%) by grade II, 4 (18%) by grade III, and 13 (59%) by grade IV stenosis. The stenosis involved one laryngeal site in 13 (59%) patients and multiple sites in 9 (41%) (Table 2).

**Table 1:** Demographic characteristics of patients

	Overall (*N* = 22)
Hospital	
Genoa	19 (86.4%)
Cagliari	3 (13.6%)
Age	
Mean (SD)	54.9 (20.4)
Median [Min, Max]	57.0 [11.0, 85.0]
Gender	
F	9 (40.9%)
M	13 (59.1%)
Etiology	
1 post-surgery	4 (18.2%)
2 post-RT	9 (40.9%)
3 post-intubation	5 (22.7%)
4 post-trauma	1 (4.5%)
5 autoimmune	1 (4.5%)
6 idiopathic	2 (9.1%)

**Table 2:** Bogdasarian classification of patients

Overall (*N* = 22)	
Bogdasarian	
1	2 (9.1%)
2	3 (13.6%)
3	4 (18.2%)
4	13 (59.1%)
Bogdasarian_IV	
I–III	9 (40.9%)
IV	13 (59.1%)

### Surgical technique

**Fig. 1:**
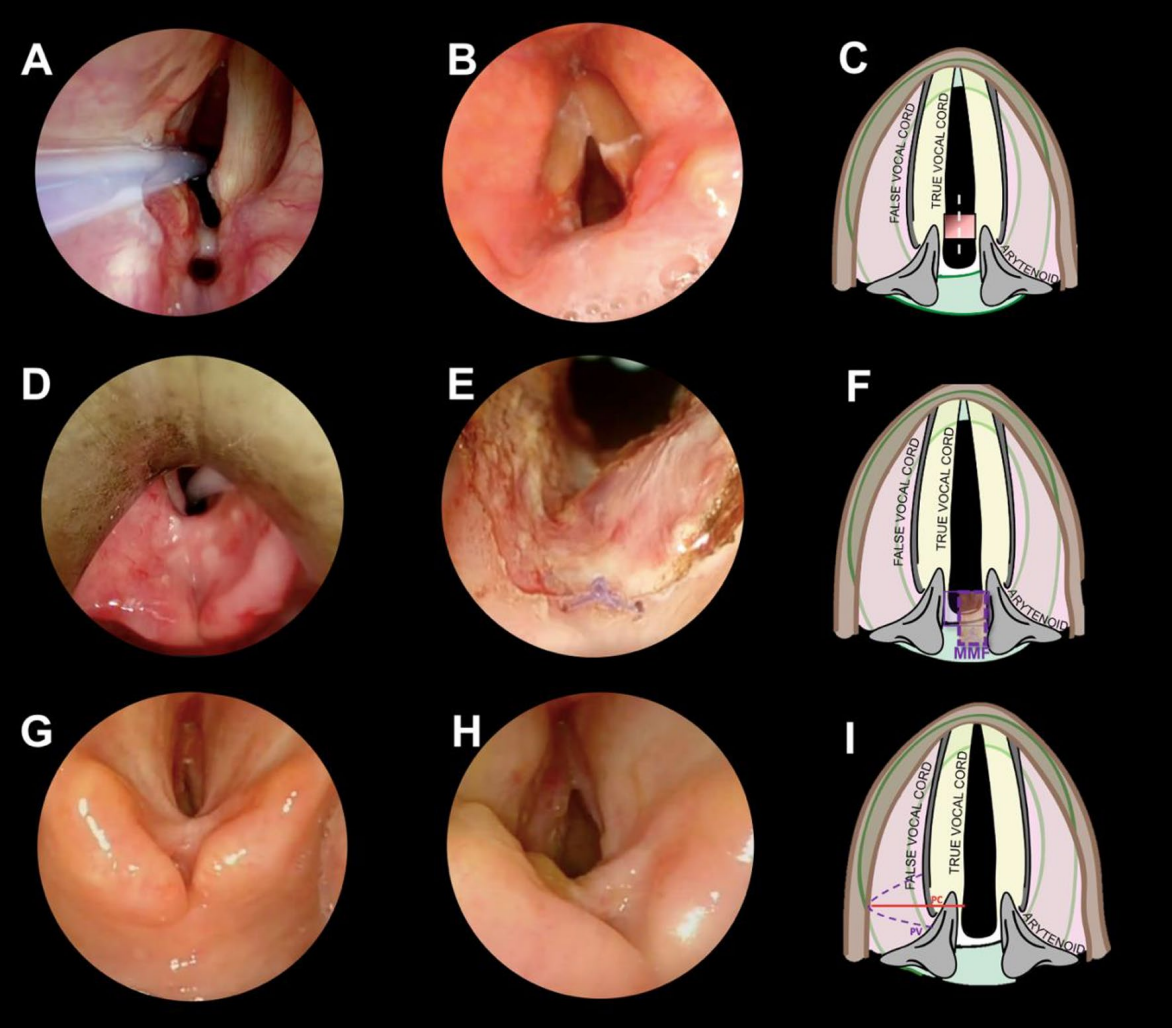
**A** A case of grade I PGS: an interarytenoid synechia with a sinus tract posteriorly. **B** 3-month follow-up endoscopic pictures following endoscopic lysis, **C** the first-choice treatment for grade I PGS is endoscopic lysis, because healthy tissue, spared between the row areas due to resection of the fibrotic bridge, avoids web reformation in the interarytenoid region. **D** A case of grade III PGS: interarytenoid and posterior commissure scarring, involving one cricoarytenoid joint. **E** Final intraoperative result of J-shaped posterior mucosal microflap for a grade 3 PGS. The microflap is pedicled inferolaterally and sutured in the posterior commissure, with a single resorbable stitch (*). By doing so we avoid the contact of 2 row surfaces, reducing the risk of restenosis. **F** Graphical representation of the J-shaped mucosal microflap. The continuous line represents the donor mucosa. The dotted line shows the final position of the inferolateral pedicled J-shaped mucosal microflap. **G** grade IV PGS: posterior commissure scarring involving both cricoarytenoid joints. **H** Post-treatment endoscopic picture of a left posterior cordotomy. (I) The surgical techniques of a left posterior cordotomy (PC). The surgical procedure began with a unilateral partial ventriculectomy (PV) to remove the posterior third of the false vocal fold to achieve adequate exposure of the posterior third of the true vocal cord and the floor of the ventricle. Afterward, a CO_2_ laser incision of the mucosa of the true vocal fold, including the vocal process, was performed. The CO_2_ laser cut was then extended through both vocalis and muscularis parts of the thyroarytenoid muscle, up to the inner perichondrium of thyroid lamina, laterally. Caudally, the section was extended up to the superior margin of cricoid.

Surgical treatment included TOLMS in terms of tailored laser resection of the scar tissue combined with posterior cordotomy in 13 cases (59.1%), resurfacing of the raw area with mucosal microflap in 4 (18.2%), and placement of a Montgomery T-tube in 4 (18.2%) or keel stent in 1 (4.5%) patient (Fig. 1).

TOLMS was performed using a CO_2_ laser (Lumenis Encore Ultrapulse, Tel Aviv, Israel) coupled with an Acublade micromanipulator set at a power of 1–3 W, delivered in an ultrapulse modality in continuous way. A customized Montgomery T-tube stent was placed at the end of the surgical procedure in 4 (18%) patients and left in place for at least 6 months to guarantee a stable airway and to avoid further synechiae formation. A laryngeal Keel stent was placed in 1 (4.5%) patient for 6 weeks.

Infraglottic high frequency jet ventilation (HFJVIG) was used instead of perioperative tracheotomy in 6 (27.3%) patients with severe stenosis and narrow glottic space, thus making the standard orotracheal intubation unsafe.

Medical adjuvant therapy at the end of surgery, in terms of the topical application of mitomycin C (at a concentration of 2 mg/ml and kept in place for 2–4 min) and local corticosteroid injections [1–2 ml of triamcinolone acetonide at a concentration of 40 mg/ml], was administered to 2 (9%) and 3 (13.6%) patients, respectively. In Table 3 we summarize our strategy for endoscopic treatment related to Bogdasarian grade.

**Table 3:** Endoscopic treatments of PGS related to Bogdasarian grade

Bogdasarian Grade	Endoscopic treatment
I	Lysis of the synechia
II	Lysis of the synechia and mucosal flap ± T-tube
III	Lysis of the synechia and mucosal flap ± posterior cordotomy ± T-tube
IV	Lysis of the synechia and posterior cordotomy ± T-tube

### Outcome evaluation and statistical analysis

All patients had been evaluated and staged both preoperatively and postoperatively at least after 6 months or at the last available follow-up (follow-up was updated until October 2020) by the Airway-Dysphonia-Voice-Swallowing (ADVS) staging system [19, 20], Voice Handicap Index-30 (VHI-30) [21, 22], and Eating Assessment Tool-10 (EAT-10) [23, 24] questionnaires.

Postoperative quality of life as tested by ADVS, VHI-30, and EAT-10 questionnaires was considered as the primary outcome. As secondary outcomes, we considered time to retreatment and time to decannulation for patients with previous tracheostomy (*N* = 12, 54.5%) at the time of the transoral surgery. Clinical data are reported with absolute and relative frequencies. Statistical analysis was performed by Mann–Whitney–Wilcoxon or Wilcoxon signed-rank tests, as appropriate. Survival analysis was performed with the Kaplan–Meier method and group comparisons by log-rank test. In all analyses, a two-tailed *p* value < 0.05 was considered significant. R (version 4.0.2) was used for statistical analysis.

## Results

Median follow-up time was 38.5 months (IQR 14.8–61.3 months). During follow-up, 5 patients (22.7%) required retreatment, of whom 4 (18.2%) needed two procedures and 1 (4.5%) a third procedure to obtain a patent and stable airway.

**Fig. 2:**
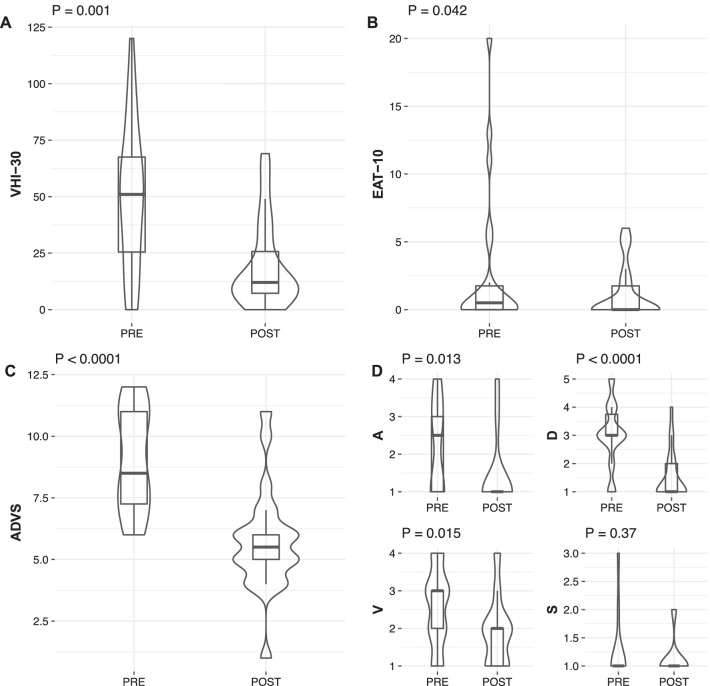
Box plots and violin plots showing the pre-treatment (PRE) and post-treatment (POST) distributions of paired results of VHI-30 (**A**), EAT-10 (**B**), and ADVS (**C**, **D**) questionnaires. *P* values estimated by Wilcoxon signed-rank test

Quality of life significantly improved as measured by the VHI-30 questionnaire, with a median variation of − 31.0 (95% CI − 48.0, − 12.0; *p* = 0.003; Fig. 2A), the EAT-10 with a median variation of − 4.0 (95% CI − 8.0, − 1.0; p = 0.042; Fig. 2B), and the ADVS with a median variation of − 3.5 (95% CI − 4.5, − 2.5; *p* < 0.001; Fig. 2C). Among the ADVS categories (Fig. 2D) significant improvement was observed for airway status with a median variation of − 2.0 (95% CI − 2.5, − 0.5; *p* = 0.012), dyspnea score with a median variation of − 2.0 (95% CI − 2.0, − 1.5; p < 0.001), and voice score with a median variation of − 1.0 (95% CI − 1.5, − 0.5; *p* = 0.015). No significant changes were observed for swallowing scores: median variation of − 0.5 (95% CI − 1.5, + 1.0; *p* = 0.37).

Twelve patients had a previous tracheostomy at the time of the surgical procedure. This group was composed of 7(58%), 2 (17%), 2 (17%), and 1(8%) patients affected by PGS grades IV, III, II, and I, respectively.

**Fig. 3:**
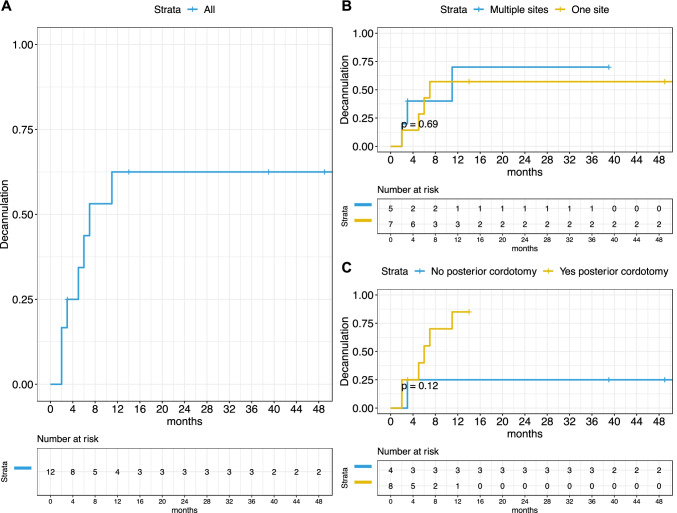
Kaplan–Meier curves showing the probability of decannulation for the whole cohort (**A**) or stratified by sites involved (**B**) or posterior cordotomy (**C**). *P* value estimated by log-rank test

**Fig. 4:**
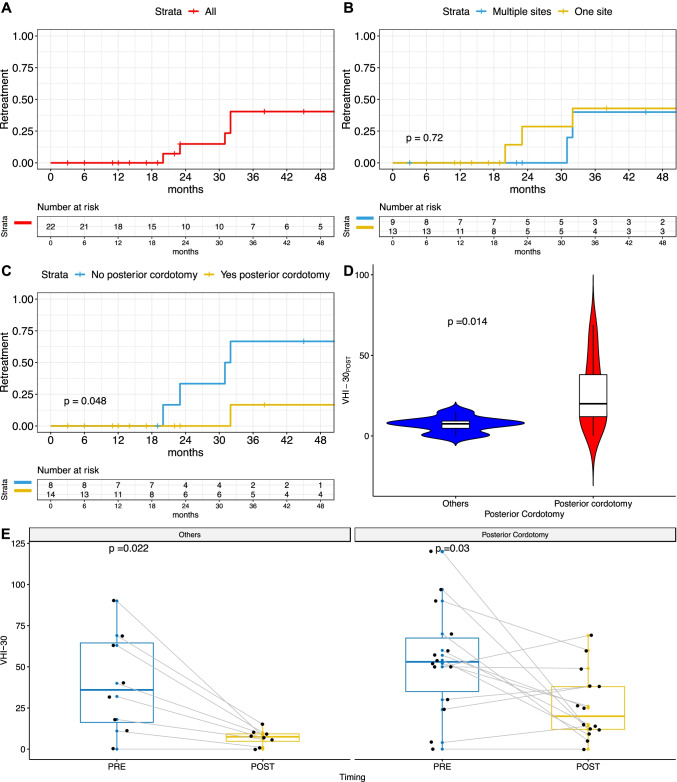
Kaplan–Meier curves showing the probability of retreatment for the whole cohort (**A**) or stratified by sites involved (**B**) or posterior cordotomy (**C**). P value estimated by log-rank test. Box plots and violin plots showing the worse postoperative VHI-30 score in patients submitted to posterior cordotomy, compared to others procedures (**D**) but the significant post-treatment improvement in both these subgroups (**E**). *P* values estimated by Mann–Whitney–Wilcoxon or Wilcoxon signed-rank tests, respectively

Among the 12 patients with a previous tracheostomy at the time of the surgical procedure, 7 were successfully decannulated (58%), 2 were still cannula dependent with narrow airway, 2 patients refused the decannulation despite a sufficient respiratory space, and 1 was lost to follow-up. In view of the above, we were able to decannulate 7 of 9 patients (almost 80%). We were not able to decannulate two of these patients, 1 had a PGS grade I and the other one a grade II. Remarkably, there was no association between low grade of PGS and the rate of decannulation. On the other hand, the choice of performing a posterior cordotomy, only guided by the presence of PGS grades IV and III, assured a high rate of decannulation: 7 out of 9 tracheostomized patients were successfully decannulated and 2 were lost to follow-up. For the entire group (Fig. 3A), the estimated decannulation probability at 6 months was 44% (95% CI 5.7–66%) reaching 62% (95% CI 19–83%) at 1 year, with no changes at longer times (3 years: 62%; 95% CI 19–83%) (Fig. 3A). Neither the involvement of more than one laryngeal site (Fig. 3B) nor the choice of the posterior cordotomy as procedure was significantly associated with a different decannulation outcome, despite a trend was observable in favor of posterior cordotomy (1-year estimate 85% vs 25%, *p* = 0.12; Fig. 3C). The estimated risk of retreatment at 1, 2, and 3 years was 0.0% (95% CI 0.0–0.0%), 15.0% (95% CI 0.0–32.0%), and 40.0% (95% CI 5.3–63.0%), respectively, as shown in Fig. 4A. The involvement of more than one laryngeal site was not associated with a different risk of retreatment (Fig. 4B). The choice of performing a posterior cordotomy, only guided by the presence of a Bogdasarian IV stenosis, was associated with a significant reduction in the risk of retreatment (Fig. 4C). Of note, in this group the post-treatment VHI-30 score was significantly worse (*p* = 0.014, Fig. 4D), despite a significant improvement was anyway observed, comparing the paired pre-treatment score (*p* = 0.03; Fig. 4E). This was also observed in the group treated without posterior cordotomy (*p* = 0.022; Fig. 4E).

Interestingly, all patients who needed a retreatment were affected by grade III PGS. Only 1 out of 5 was treated with posterior cordotomy as upfront treatment, to underline the importance of a more extensive intervention when indicated. Four of them (80%) were successfully treated with a further transoral laser CO_2_ procedure, and 1 (20%) with a tracheotomy, because he refused any other endoscopic treatment.

## Discussion

PGS is a complex and challenging pathology, ranging from a simple interarytenoid fibrotic bridge to fixation of both cricoarytenoid joints with the vocal cords fixed in a paramedian or median position in all cases. Atallah et al. [25] modified the Bogdasarian classification system by introducing the subdivisions of grades III and IV into two subcategories IIIa, IIIb, and IVa, IVb, respectively, underlining the key role of the involvement and fixation of the joint on the decision-making process and final outcome.

The correct choice of the surgical option at the first attempt is of paramount importance and depends on adequate and comprehensive airway evaluation and careful selection of the patient.

There are no doubts that transoral lysis of the scar is an adequate treatment for mild PGS (Grades I-II) (Fig. 4), because the interposition of healthy tissue between the raw areas avoids interarytenoid web reformation. Even in our series, the two patients affected by grade I PGS were successfully treated by simple endoscopic lysis.

On the other hand, compound laryngoplasty interventions have been described for patients affected by more severe PGS [9, 10]. Moreover, in the case of PGS extending to the subglottis, data from the literature suggest the need for more complex surgeries, in terms of widening the airways with cartilaginous grafts [1, 17].

In 2004, Rutter and Cotton [26] reported a 97% decannulation rate in a cohort of 29 patients with PGS when placing a costal cartilaginous graft but with the need of an open approach through a laryngofissure in all patients.

Montgomery et al. [12] firstly described a postcricoid mucosal advancement flap (PCMAF) through a laryngofissure. More recently, combined glottic reconstruction (CGR) [15] has been described to overcome poor access or unfavorable cricoid anatomy during PCMAF procedures. It is performed via a laryngofissure and consists of a posterior cricoid split with cartilage grafting, remobilization of the cricoarytenoid joints, and advancement of a vascularized PCMAF to achieve primary posterior glottic wound closure. Unfortunately, in case of open-neck surgery, a tracheotomy is always required to protect the airway during the postoperative period. It is well known that a tracheostomy has deleterious effects on voice and swallowing, and may increase the risk of other morbidities, such as acute airway obstruction, secondary stenosis, and even injury to large vessels [4]. As a consequence, following open approaches, decannulation rates can decrease to 70% or less, making 30% of cases tracheostomy dependent when they were originally free of a tracheostomy [27]. Moreover, laryngofissure can lead to nonunion of the thyroid lamina, chondritis, and scarring of the anterior commissure with anterior glottic web formation causing permanent hoarseness and airway obstruction [4].

To overcome the complications related to open approaches, in 2010 Goldberg et al. [28] were the first to outline the transoral PCMAF in 2 patients with grade II PGS. Atallah et al. [4] firstly described the using of PCMAF for Bogdasarian grades III and IV, with a rate of decannulation of 100%. To date, the decannulation rate reported in the literature harvesting the PCMAF through a transoral approach ranges from 91 to 100% in grades II–IV [1].

Moreover, the technique of transoral placement of the costal grafting in the split plate of the cricoid (EPCS/RG) showed an excellent decannulation rate of 100% for children affected by grade III PGS, with a slight decrease to 90% in children with grade IV PGS [1].

Our series was mainly constituted by patients with severe PGS (grade IV 59%). In all these cases, a wider opening of the posterior glottic area was achieved by posterior cordotomy, in addition to laser re-modelling of the stenotic area involving the posterior commissure (Fig. 1G–I). The addition of the posterior cordotomy allowed for a significant reduction of the risk of retreatment, without the need for an open-neck approach by laryngofissure in all cases.

Our results showed a decannulation rate of nearly 80%, which is comparable with those reported in the literature [1, 4].

Moreover, from a functional point of view, we observed improvement of voice-related quality of life, with a significant reduction in VHI-30 (*p* = 0.003) and the voice item of the ADVS (*p* = 0.015), despite a wider opening due to the posterior cordotomy performed in 13 patients. The unexpected improvement of vocal function could be related to mitigation of long-term dyspnea related to air trapping between the adducted vocal folds, with subsequent breathlessness and difficult phonatory coordination [29, 30].

Regarding swallowing, no significant worsening was observed postoperatively even in case of wider opening with a posterior cordotomy. This result is probably related to fact that if the approximation of false vocal folds and arytenoids to epiglottic petiole is completely maintained and efficient, the airway protection may be valuable even if the glottic closure is incomplete [31]. In fact, sphincter function is the result of a more complex physiology where the integrity of the TA muscles represents only a part of it.

In conclusion, even if there is still no general agreement on an exact algorithm to treat PGS, our results confirm that transoral surgery in terms of scar tissue removal with or without posterior cordotomy, combined in selected patients with pedicled local flaps and/or stents, is a safe and effective surgical approach even for more severe PGS. According to our experience, more advanced grade of PGS, required a more aggressive upfront treatment, reducing the risk of recurrence. Hence, according to our data, there are no absolute contraindication to transoral surgery for each grade of PGS. Moreover, these transoral procedures do not preclude any further more extensive open treatment, avoiding in most cases the need of perioperative or even permanent tracheotomy.

From a functional point of view, TOLMS offers significant improvement of the quality of life assuring acceptable respiratory function with the removal of the cannula in most tracheotomized patients. Moreover, unexpected subjective improvement of voice is frequently reached in most patients. The deterioration of vocal outcomes is directly correlated with the entity of the glottic opening, worsening in cases of large incompetence. However, the maintenance of both arytenoids guarantees an efficient sphincteric function, without impairment swallowing ability even in case of posterior cordotomy and respiratory space augmentation.
